# *PNPLA3* genotype and fibrosis-4 index predict cardiovascular diseases of Japanese patients with histopathologically-confirmed NAFLD

**DOI:** 10.1186/s12876-021-02020-z

**Published:** 2021-11-19

**Authors:** Norio Akuta, Yusuke Kawamura, Yasuji Arase, Satoshi Saitoh, Shunichiro Fujiyama, Hitomi Sezaki, Tetsuya Hosaka, Masahiro Kobayashi, Mariko Kobayashi, Yoshiyuki Suzuki, Fumitaka Suzuki, Kenji Ikeda, Hiromitsu Kumada

**Affiliations:** 1 Department of Hepatology, Toranomon Hospital and Okinaka Memorial Institute for Medical Research, 2-2-2 Toranomon, Minato-ku, Tokyo, 105-8470 Japan; 2 Liver Research Laboratory, Toranomon Hospital, Tokyo, Japan

**Keywords:** Nonalcoholic fatty liver disease, Nonalcoholic steatohepatitis, Cardiovascular diseases, Malignancies, Liver-related events, FIB-4 index, *PNPLA3*

## Abstract

**Background:**

Reliable noninvasive predictors of the top three causes of death [cardiovascular diseases (CVDs), malignancies, and liver-related events in patients with non-alcoholic fatty liver disease (NAFLD)] have not yet been determined.

**Methods:**

We retrospectively investigated the incidence of three complications [CVDs, malignancy (except for liver cancer), and liver-related events] in 477 Japanese patients with histo-pathologically confirmed NAFLD for a median follow-up of 5.9 years. In addition to histological findings, we also investigated noninvasive predictors.

**Results:**

A score of ≥ 2.67 for the noninvasive diagnosis of stage 4 fibrosis based on the Fibrosis-4 (FIB-4) index indicated a high level area under the receiver operating characteristic (AUROC) curve (0.90), sensitivity (82.9%), specificity (86.4%), and negative predictive value [(NPV) of 98.5%]. The yearly incidence rates of CVDs, malignancies, and liver-related events were found to be 1.04%, 0.83%, and 0.30%, respectively. Multivariate analysis identified a FIB-4 index ≥ 2.67 score as a significant and independent, noninvasive predictor of these three complications. Furthermore, the cumulative incidence rates of CVDs were significantly different among the three genotypes of *PNPLA3*. *PNPLA3* genotype CC, chronic kidney disease (CKD), and FIB-4 index ≥ 2.67 was could be attributed to these three significant CVD risk factors. The rates of CVDs were significantly different among the three subgroups based on the combination of risk factors. In malignancy (except for liver cancer), the incidence rate of colon cancer was 25.0%; in particular, the rate in females was 53.8%.

**Conclusions:**

Our results highlighted the importance of the *PNPLA3* genotype and FIB-4 index ≥ 2.67 on the incidence of complications in Japanese patients with NAFLD, especially the incidence of CVDs. Early diagnosis, based on the presence of one or more risk factors, and early treatment might improve the prognosis for NAFLD patients.

## Background

The most common worldwide liver disease is non-alcoholic fatty liver disease (NAFLD) [1–6]. Its pathology ranges from the typically benign non-alcoholic fatty liver to non-alcoholic steatohepatitis (NASH), which may progress to liver cirrhosis, liver cancer, and finally, to liver failure [7].

The American Association for the Study of Liver Diseases reported that the most common cause of death in patients with NAFLD is related to cardiovascular diseases (CVDs) independent of other metabolic comorbidities. Liver-related mortality was reported to be the second or third cause of death, and cancer-related mortality was among the top three causes of death [8]. In Asia, the incidence rates of CVDs, malignancies, and liver-related events in patients with histo-pathologically confirmed NAFLD still remains unclear.

Previous studies have suggested that the stage of fibrosis is a more reliable predictor of liver-specific mortality than the NAFLD activity score (NAS) [9]. The stage of fibrosis, exclusive of other histopathological features of steatohepatitis, has been reported to be an independent and significant predictor of overall mortality, need for liver transplantation, and liver-related events [10]. Furthermore, the fibrosis stage was also associated with the CVD incidence [11, 12]. However, reliable, non-histological, and noninvasive predictors of the top three causes of death have not yet been found.

The purpose of the present study was to determine the incidence rates of three complications [CVDs, malignancies (except for liver cancer), and liver-related events as the top three causes of death] and noninvasive predictors of these complications in patients with NAFLD by retrospectively analyzing the outcome of 477 Japanese patients with histo-pathologically confirmed NAFLD.

## Methods

### Patients

This study was designed as a retrospective cohort study of patients with histo-pathologically-confirmed NAFLD. Between 1976 and 2021, liver biopsies were performed at our hospital for patients with liver dysfunction and/or fatty liver diagnosed by abdominal ultrasonography. Of those conditions, the diagnosis of NAFLD was confirmed in 477 patients based on histopathology. Patient characteristics at the time of histopathological diagnosis of NAFLD are summarized in Table 1.

**Table 1:** Patient characteristics at the time of histological diagnosis of non-alcoholic fatty acid liver disease (NAFLD)

*Demographic data*	
Numbers of patients	477
Gender, male/female, n	282/195
Age, year*	53 (20–87)
Body mass index, kg/m^2^*	26.3 (16.6–42.4)
Waist circumference, cm*	91.3 (68.2–132.1)
Smoking, absence/presence, n	385 /92
*Previous or current events*	
Cardiovascular diseases, absence/presence/unknown, n	444/30/3
Malignancies, except for liver cancer, absence/presence/unknown, n	426/47/4
Liver-related events, absence/presence/unknown, n	432/42/3
Liver cancer, absence/presence/unknown, n	446/30/1
Ascites, absence/presence/unknown, n	472/2/3
Hepatic encephalopathy, absence/presence/unknown, n	474/0/3
Jaundice, absence/presence/unknown, n	471/3/3
Esophago-gastric varices, absence/presence/unknown, n	463/11/3
*Comorbid diseases*	
Type 2 diabetes mellitus, absence/presence, n	321/156
Hypertension, absence/presence, n	256/221
Dyslipidemia, absence/presence, n	125/352
Hyperuricemia, absence/presence, n	420/57
Chronic kidney disease, absence/presence, n	445/32
*Histological findings*	
Steatosis, 5–33%/ > 33–66%/ > 66%, n	174/175/126
Lobular inflammation	
No foci/< 2 foci/2–4 foci/> 4 foci per 200 × field, n	28/263/167/17
Ballooning, none/few cells/many cells, n	42/298/135
Fibrosis stage, 0/1/2/3/4, n	55/189/77/121/35
NAFLD activity score, ≤ 2/3/4/ ≥ 5, n	38/197/242
*Genetic factors*	
*PNPLA3* rs738409 (CC/CG/GG/Not determined)	57/137/135/148
*TM6SF2* rs58542926 (CC/CT/TT/Not determined)	248/74/7/148
*ALDH2* rs671 (GG/GA/AA/Not determined)	158/142/29/148
*HSD17B13* rs6834314 (AA/AG/GG/Not determined)	162/132/25/158
*Laaboratory data*^a^	
Serum aspartate aminotransferase (AST), U/L	44 (3–378)
Serum alanine aminotransferase (ALT), U/L	69 (13–783)
Gamma-glutamyl transpeptidase, U/L	71 (11–1,135)
Platelet count, × 10^3^/mm^3^	212 (40–471)
Albumin	4.1 (2.8–6.9)
Serum ferritin, μg/L	227 (< 10–2.067)
High sensitive C-reactive protein, mg/dL	0.097 (0.004–2.240)
FIB-4 index	1.36 (0.19–14.8)

Data represent number of patients, except those denoted by ^a^, which represent the median (range) values

Cardiovascular diseases included coronary artery disease, heart valve disease, arrhythmia, heart failure, hypertension, orthostatic hypotension, shock, endocarditis, diseases of the aorta and its branches, disorders of the peripheral vascular system, and stroke

Based on the practice guidance from the American Association for the Study of Liver Diseases [8], NAFLD diagnosis was based on the liver histopathological findings of steatosis in ≥ 5% of hepatocytes after excluding other liver diseases (such as autoimmune hepatitis, primary biliary cholangitis, viral hepatitis, drug-induced liver disease, biliary obstruction, hemochromatosis, Wilson disease, and α-1-antitrypsin deficiency-associated liver disease). Of these 447 patients, none of them who consumed more than 20 g of alcohol per day.

The Human Ethics Review Committee at Toranomon Hospital approved the protocol of the study (number 953), and a signed informed consent form was obtained from each of the patients at the time of liver histological diagnosis. The study complied with the International Conference on Harmonization Guidelines for Good Clinical Practice (E6), as well as the 2013 Declaration of Helsinki.

### Diagnosis and follow-up

Liver-related events were evaluated as jaundice, ascites, esophago-gastric varices, hepatic encephalopathy, and liver cancer. CVDs included heart failure, coronary artery disease, hypertension, orthostatic hypotension, shock, heart valve disease, endocarditis, arrhythmia, disorders of the peripheral vascular system, diseases of the aorta and its branches, and stroke [6]. After the NAFLD diagnosis, biochemical and hematological data were collected at least twice yearly. At least once annually, abdominal ultrasonography (US), computed tomography (CT), or magnetic resonance imaging (MRI) studies were performed [6]. In all of 477 patients, the median duration of follow-up from diagnosis to the last visit or death was 5.9 years (range 0.0–44.9 years).

### Liver histopathology

Liver specimens were obtained with a 14-gauge modified Vim Silverman needle (Tohoku University style, Kakinuma Factory, Tokyo, Japan), a 16-gauge core tissue biopsy needle (Bard Peripheral Vascular Inc., Tempe, AZ) or surgical resection. Specimen was fixed in 10% formalin, and the prepared sections were stained with hematoxylin–eosin, Masson trichrome, silver impregnation, or periodic acid-Schiff after diastase digestion. An adequate liver biopsy sample was defined as a specimen longer than 1.5 cm and/or containing more than 11 portal tracts [6]. Four pathologists (K.K., F.K., T.F., and T.F.), who were blinded to the clinical findings, evaluated each specimen, and the final assessment was reported by consensus [6].

Steatosis grades 0, 1, 2, and 3 corresponded to hepatocyte steatosis levels of < 5%, ≥ 5– < 33%, ≥ 33– < 66%, and ≥ 66%, respectively. Lobular inflammation scores of 0, 1, 2, and 3 corresponded to no, < 2, 2–4, and ≥ 4 foci per 200 × field, respectively. Hepatocyte ballooning scores of 0, 1, and 2 corresponded to none, few, and many cells, respectively. The sum of the steatosis, lobular inflammation, and hepatocyte ballooning scores (range 0–8 points) is termed the NAS [13]. Fibrosis stage was defined as 0, 1, 2, 3, or 4 [13, 14].

### Clinical parameters

The fibrosis-4 (FIB-4) index has been used as a parameter for fibrosis progression [15]. The FIB-4 index is useful for excluding NAFLD with advanced fibrosis based on values < 1.30, which are considered to represent non-advanced fibrosis. and a FIB-4 index ≥ 2.67 is suggested before performing a liver biopsy to identify advanced fibrosis [5]. Chronic kidney disease (CKD) was defined as persistent positive proteinuria and/or estimated glomerular filtration rate (eGFR) < 60 ml/min/1.73 m^2^ for more than three months [16]. Normal waist circumferences were defined as 85 and 90 cm for men and women, respectively.

The TaqMan SNP genotyping assay (Applied Biosystems, Foster City, CA, USA) was used for genotyping *PNPLA3* rs738409, *TM6SF2* rs58542926, *ALDH2* rs671, and *HSD17B13* rs6834314.

### Statistical analysis

The incidence of each event was analyzed during the period from the time of histopathological NAFLD diagnosis until the last visit or occurrence of event. Stepwise Cox regression analysis was used to determine independent predictive factors associated with the incidence of CVDs, malignancies (except for liver cancer), and liver-related events. The hazard ratio and 95% confidence interval (HR and 95% CI, respectively) were also calculated. The potential predictive factors included several noninvasive variables, except for some confounding factors and histological findings: (1) demographic data (except for age), (2) previous and/or current events, (3) comorbid diseases, (4) genetic factors, and (5) laboratory data [except for aspartate and alanine transaminases (AST and ALT, respectively) and platelet count] as shown in Table 1. Variables that were statistically significant based on univariate analysis were tested using a multivariate analysis to identify significant independent factors after being converted into categorical data consisting of two simple ordinal numbers. Significance was set at *P* value < 0.05 by the two-tailed test. Statistical comparisons were performed with the SPSS software (SPSS Inc., Chicago, IL, USA). The area under the receiver-operating characteristic curve (AUROC), sensitivity, specificity, positive and negative predictive values (PPV and NPV, respectively) were calculated to determine the diagnostic performance of FIB-4 index for liver fibrosis detection.

## Results

### Diagnostic performance of FIB-4 index score for detection of liver fibrosis in NAFLD

Data from 477 patients were used for analysis of the diagnostic performance of FIB-4 index for the detection of liver fibrosis in NAFLD patients. The areas under the AUROC of the FIB-4 index for detection of stages ≥ 1, ≥ 2, ≥ 3, and 4 were 0.75 (95% CI 0.69–0.81), 0.84 (95% CI 0.80–0.87), 0.85 (95% CI 0.81–0.88), and 0.90 (95% CI 0.87–0.94), respectively. Sensitivity, of FIB-4 index ≥ 1.30 for detection of stages ≥ 1, ≥ 2, ≥ 3, and 4 were 56.4%, 78.2%, 95.2%, and 18.9%, respectively. Specificity values were 79.8%, 73.8%, 74.4%, and 79.3%, respectively; PPV values were 88.5%, 65.1%, 55.2%, and 92.1, respectively while NPV values were 100%, 51.4%, 14.0%, and 100%, respectively. Sensitivity results of the FIB-4 index ≥ 2.67 for detection of stages ≥ 1, ≥ 2, ≥ 3, and 4 were 20.9%, 98.2%, 98.9%, and 13.9%, respectively. Specificity values were 36.1%, 98.0%, 94.4%, and 61.6%, respectively. PPVs were 46.2%, 94.7%, 80.9%, and 78.4%, respectively while NPV values were 82.9%, 86.4%, 32.6%, and 98.5%, respectively. Thus, a FIB-4 index ≥ 2.67 for the noninvasive diagnosis of stage 4 indicated high AUROC, sensitivity, specificity, and NPV values (Table 2).

**Table 2:** Diagnostic performance of Fibrosis-4 (FIB-4) index for the detection of liver fibrosis

Overall (n = 477)	AUROC	(95%CI)	Fib-4 cut off	Sensitivity (%)	Specificity (%)	PPV (%)	NPV (%)
Fibrosis stage 1–4 (n = 422) versus Fibrosis stage 0 (n = 55)	0.75	(0.69–0.81)	1.30	56.4	78.2	95.2	18.9
			2.67	20.9	98.2	98.9	13.9
Fibrosis stage 2–4 (n = 233) versus Fibrosis stage 0–1 (n = 244)	0.84	(0.80–0.87)	1.30	79.8	73.8	74.4	79.3
			2.67	36.1	98.0	94.4	61.6
Fibrosis stage 3–4 (n = 156) versus Fibrosis stage 0–2 (n = 321)	0.85	(0.81–0.88)	1.30	88.5	65.1	55.2	92.1
			2.67	46.2	94.7	80.9	78.4
Fibrosis stage 4 (n = 35) versus Fibrosis stage 0–3 (n = 442)	0.90	(0.87–0.94)	1.30	100	51.4	14.0	100
			2.67	82.9	86.4	32.6	98.5

AUROC, area under the receiver-operating characteristic curve; CI, confidence interval; PPV, positive predictive value; NPV, negative predictive value

### Incidence and noninvasive predictors of CVDs in NAFLD

**Fig. 1:**
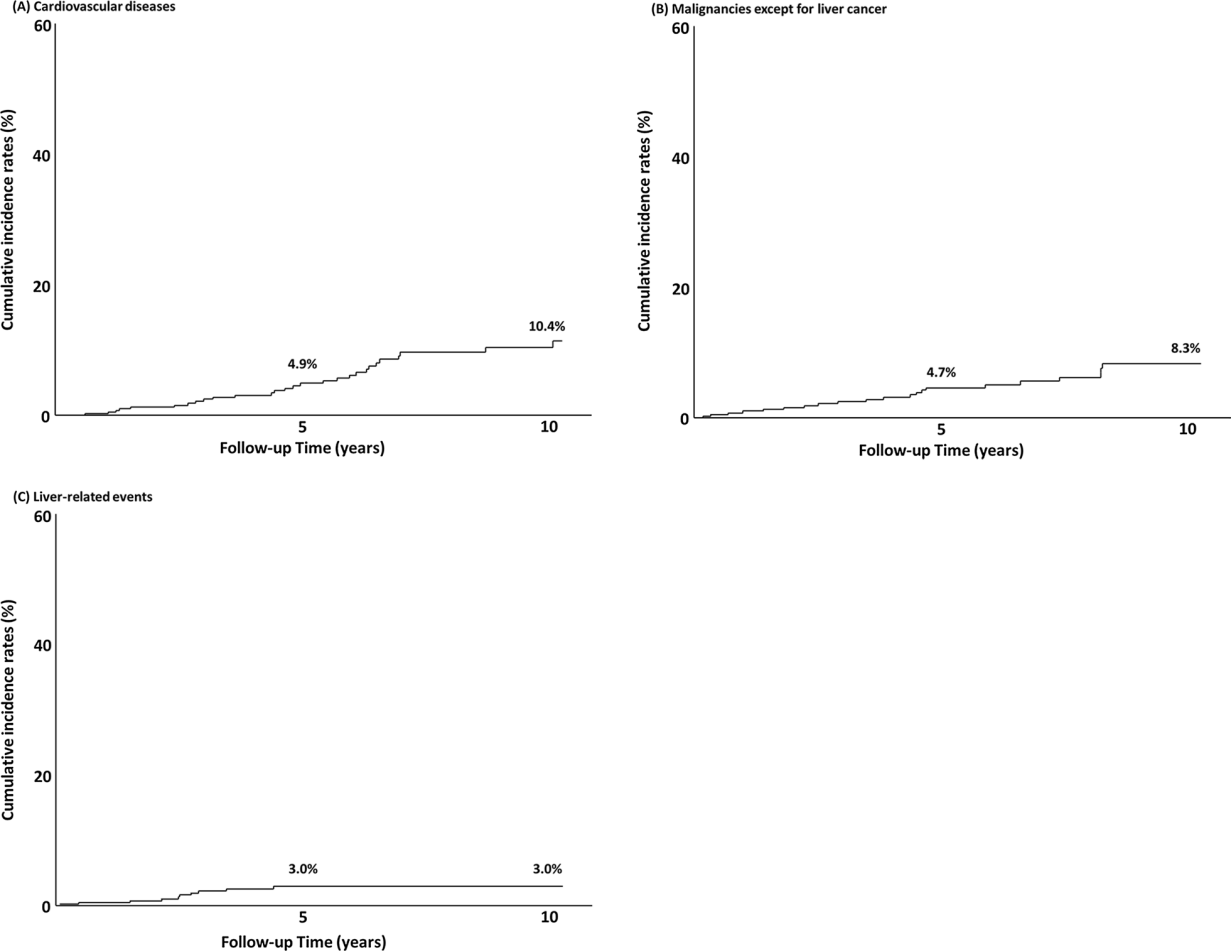
Cumulative incidence rates of three complications in Japanese patients with non-alcoholic fatty liver disease (NAFLD). **A** The yearly incidence of cardiovascular diseases (CVDs) was 1.04%. **B** The yearly incidence of malignancies except for liver cancer was 0.83%. **C** The yearly incidence of liver-related events was 0.30%

Characteristics of the 444 patients who were confirmed to have no previous or current CVDs at their NAFLD diagnoses were evaluated for the rate of CVD development. During the follow-up period, 43 patients (9.7%) developed CVDs. The cumulative incidence rates were 4.9% and 10.4% at the end of 5 and 10 years, respectively. The yearly incidence rate of CVDs over the 10-year period under study was 1.04% (Fig. 1a).

Univariate analysis identified six noninvasive parameters that significantly correlated with the incidence of CVDs: (1) body mass index (BMI), (2) previous or current malignancies (except for liver cancer), (3) CKD, (4) comorbid hypertension, (5) *PNPLA3* genotype, and (6) FIB-4 index. These factors were entered into a multivariate analysis, which identified three noninvasive factors that significantly and independently influenced the incidence of CVDs: (1) *PNPLA3* genotype (CC type; HR 3.66, 95% CI = 1.63–8.35; *P* = 0.002), (2) CKD (Presence; HR 3.62, 95% CI = 1.18–11.2; *P* = 0.025), and (3) FIB-4 index (≥ 2.67; HR 2.73, 95% CI = 1.21–6.14; *P* = 0.016) as shown in Table 3.

**Table 3:** Non-invasive predictors associated with the incidence of cardiovascular diseases (CVDs)

Factor	Category	Univariate			Multivariate		
		Hazard ratio	(95% CI)	*P* value*	Hazard ratio	(95% CI)	*P* value*
*PNPLA3* rs738409	CG, GG	1			1		
	CC	2.97	(1.35–6.53)	0.007	3.66	(1.61–8.35)	0.002
Chronic kidney disease**	Absence	1			1		
	Presence	4.44	(1.69–11.7)	0.002	3.62	(1.18–11.2)	0.025
FIB-4 index	< 2.67	1			1		
	≥ 2.67	3.14	(1.58–6.23)	0.001	2.73	(1.21–6.14)	0.016
Body mass index	< 25.0 kg/m^2^	1					
	≥ 25.0 kg/m^2^	2.32	(1.16–4.64)	0.017			
Previous or current malignancies, except for liver cancer	Absence	1					
	Presence	2.67	(1.11–6.42)	0.029			
Hypertension	Absence	1					
	Presence	2.20	(1.18–4.10)	0.013			

*Significance was determined using the Cox proportional hazard model. Variables that were statistically significant on univariate analysis were entered into multivariate analysis to identify significant independent factors

**Chronic kidney disease was defined as persistent positive proteinuria and/or eGFR < 60 ml/min/1.73 m^2^ for more than 3 months. Characteristics of the 444 patients who were confirmed to have no previous or current CVDs at their NAFLD diagnoses were evaluated for the rate of CVD development. During the follow-up period, 43 patients developed CVDs. CVDs included coronary artery disease, heart valve disease, arrhythmia, heart failure, hypertension, orthostatic hypotension, shock, endocarditis, diseases of the aorta and its branches, disorders of the peripheral vascular system, and stroke.

### Cumulative incidence rates of CVDs to a combination of risk factors

**Fig. 2:**
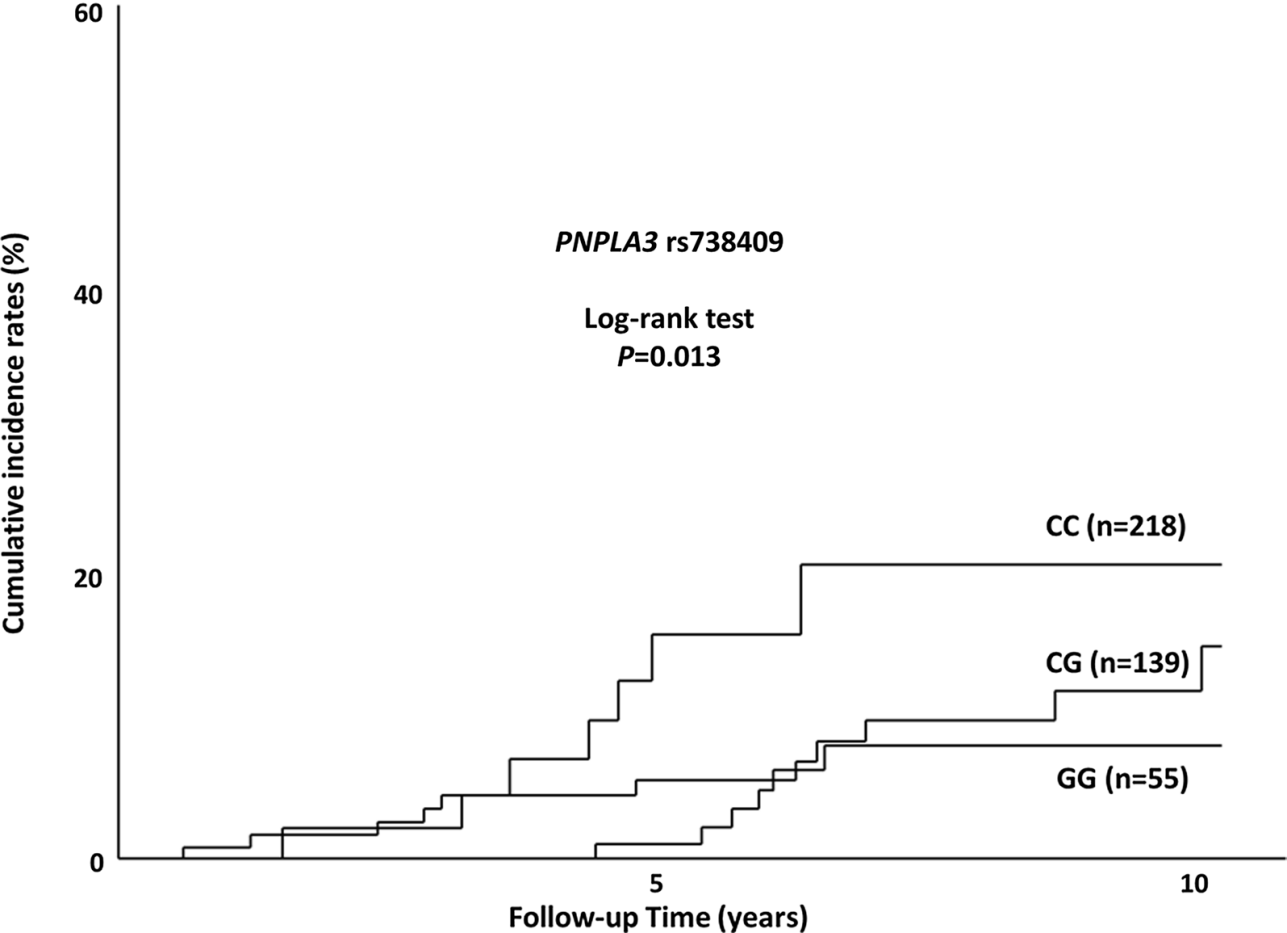
Cumulative incidence rates of cardiovascular diseases (CVDs) according to *PNPLA3* genotype. The rates were significantly different among the three genotypes (CC, CG, and GG type; *P* = 0.013; log-rank test). In particular, the rates of CC type were significantly higher than those of CG and GG types (*P* = 0.049; log-rank test type and *P* = 0.002; log-rank test, respectively). The rates of CG type were not different from those of GG type (*P* = 0.400; log-rank test)

Four-hundred twelve patients, who were confirmed to have no previous or current CVDs NAFLD diagnosis, underwent analysis to determine the cumulative incidence rates of CVDs according to *PNPLA3* genotype. Cumulative incidence rates were significantly different among the three genotypes of *PNPLA3* [CC, CG, and GG (n = 218, 139, and 55, respectively); *P* = 0.013 based on log-rank test] as shown in Fig. 2. In particular, the rates of the CC type were significantly higher than those of CG type (*P* = 0.049; log-rank test) and GG type (*P* = 0.002; log-rank test). The rates of the CG type were not different from those of the GG type (*P* = 0.400; log-rank test).

**Fig. 3:**
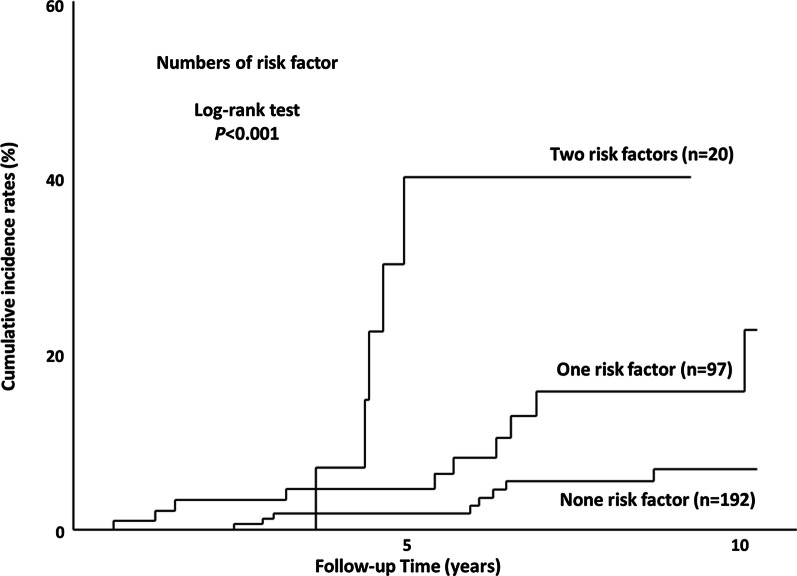
Cumulative survival rates according to the number of risk factors for CVDs. Cumulative survival rates according to the number of risk factors for CVDs, including *PNPLA3* genotype CC, presence of chronic kidney disease (CKD), and Fibrosis-4 (FIB-4) index ≥ 2.67. The cumulative incidence rates were significantly different among the three subgroups, based on the number of risk factors *(P* < 0.001; Log-rank test). Subgroup with 3 risk factors was nobody in the present study

Three-hundred nine patients, who were evaluated based on the three CVD risk factors were analyzed to determine the cumulative incidence rates according to a combination of risk factors. The cumulative incidence rates were significantly different among the three subgroups based on the number of risk factors [no, one, two, or three risk factors (corresponding to n = 192, 97, 20, or 0); *P* < 0.001] based on the log-rank test, Fig. 3. In particular, the incidence rates for patients with two risk factors were significantly higher than in those with one risk factor (*P* = 0.018; log-rank test) and no risk factors (*P* < 0.001; log-rank test). Furthermore, the incidence rates for patients with one risk factor were also significantly higher than those for no risk factors (*P* = 0.002; log-rank test).

### Incidence and noninvasive predictors of malignancies (except for liver cancer) in NAFLD

The characteristics of the 426 patients confirmed to have no previous or current malignancies (except for liver cancer) at NAFLD diagnosis were evaluated for the rate of development of malignancies (except for liver cancer). During the follow-up period, 32 patients (7.5%) developed malignancies (except for liver cancer). The cumulative incidence rates were 4.7% and 8.3% at the end of 5 and 10 years, respectively. The yearly incidence rates of malignancies (except for liver cancer) over the investigated 10-year period were 0.83% (Fig. 1b). Of the 32 patients, eight (25.0%) patients were diagnosed with colon cancer, five (15.6%) with lung cancer, four (12.5%) with pancreatic cancer, three (9.4%) with gastric cancer, three (9.4%) with prostate cancer, and nine (28.1%) with other cancers (Table 4). Especially, five of 19 males (26.3%) were diagnosed with lung cancer, and seven of the 13 females (53.8%) were diagnosed with colon cancer.

**Table 4:** Incidence of malignancies, except for liver cancer

	Total (n = 32)	Male (n = 19)	Female (n = 13)
Colon cancer	8	1	7
Lung cancer	5	5	0
Pancreas cancer	4	3	1
Gastric cancer	3	2	1
Prostate cancer	3	3	0
Breast cancer	2	0	2
Uterine cancer	2	0	2
Malignant lymphoma	2	2	0
Esophageal cancer	1	1	0
Renal cancer	1	1	0
Bladder cancer	1	1	0

Univariate analysis identified six noninvasive parameters that correlated significantly with the incidence of malignancies except for liver cancer: (1) waist circumference, (2) previous or current liver-related events, (3) type 2 diabetes mellitus, (4) *ALDH2*, (5) *PNPLA3*, and (6) FIB-4 index. These factors were entered into multivariate analysis, which identified one noninvasive factor that significantly and independently influenced the incidence of malignancies (except for liver cancer), namely, the FIB-4 index (≥ 2.67; HR 25.2, 95% CI = 2.81–226; *P* = 0.004) as shown in Table 5.

**Table 5:** Non-invasive predictors associated with the incidence of malignancies, except for liver cancer

Factor	Category	Univariate			Multivariate		
		Hazard ratio	(95% CI)	*P* value*	Hazard ratio	(95% CI)	*P* value*
FIB-4 index	< 2.67	1			1		
	≥ 2.67	2.51	(1.10–5.74)	0.029	25.2	(2.81–226)	0.004
Waist circumference**	Small	1					
	Large**	5.78	(1.09–30.3)	0.039			
*ALDH2* rs671	GG	1					
	AA, AG	5.46	(1.82–16.4)	0.002			
*PNPLA3* rs738409	CC, CG	1					
	GG	3.64	(1.41–9.44)	0.008			
Previous or current liver-related events	Absence	1					
	Presence	3.42	(1.14–10.3)	0.028			
Type 2 diabetes mellitus	Absence	1					
	Presence	2.49	(1.19–5.21)	0.015			

*Significance was determined using the Cox proportional hazard model. Variables that were statistically significant on univariate analysis were entered into multivariate analysis to identify significant independent factors

**Large waist circumference was defined as ≥ 85 cm in men and ≥ 90 cm in women. The characteristics of the 426 patients confirmed to have no previous or current malignancies except for liver cancer at NAFLD diagnosis were evaluated for the rate of development of malignancies except for liver cancer. During the follow-up period, 32 patients developed malignancies except for liver cancer. Liver-related events included liver cancer, hepatic encephalopathy, esophago-gastric varices with bleeding, ascites, and jaundice

### Incidence and noninvasive predictors of liver-related events in NAFLD

The characteristics of the 432 patients who were confirmed to have no previous or current liver-related events at NAFLD diagnosis were evaluated for the rate of development of liver-related events. During the follow-up period, 13 patients (3.0%) developed liver-related events. The cumulative incidence rates were 3.0% and 3.0% at the end of 5 and 10 years, respectively. The yearly incidence rate of liver-related events over the 10-year study period was 0.30% (Fig. 1c). Of the 13 patients, eight (61.5%) developed liver cancer, five (38.5%) esophago-gastric varices, three (23.1%) hepatic encephalopathy, one (7.7%) developed ascites, and one (7.7%) developed jaundice. Of the 13 patients, four patients had several of the above-listed conditions.

Univariate analysis identified two noninvasive parameters that correlated significantly with the incidence of liver-related events: (1) gamma-glutamyl transpeptidase and (2) FIB-4 index. These factors were entered into multivariate analysis, which identified two noninvasive factors that significantly and independently influenced the incidence of liver-related events: (1) FIB-4 index (≥ 2.67; HR 23.2, 95% CI = 5.66–95.1, *P* < 0.001) and (2) gamma-glutamyl transpeptidase (≥ 219 U/L; HR 5.53, 95% CI = 1.70–18.0; *P* = 0.004) as shown in Table 6.

**Table 6:** Non-invasive predictors associated with the incidence of liver-related events

Factor	Category	Univariate			Multivariate		
		Hazard ratio	(95% CI)	*P* value*	Hazard ratio	(95% CI)	*P* value*
FIB-4 index	< 2.67	1			1		
	≥ 2.67	17.5	(4.49–67.8)	< 0.001	23.2	(5.66–95.1)	< 0.001
Gamma-glutamyl transpeptidase	< 219 U/L	1			1		
	≥ 219 U/L	3.45	(1.11–10.7)	0.032	5.53	(1.70–18.0)	0.004

*Significance was determined using the Cox proportional hazard model. Variables that were statistically significant on univariate analysis were entered into multivariate analysis to identify significant independent factors. The characteristics of the 432 patients who were confirmed to have no previous or current liver-related events at NAFLD diagnosis were evaluated for the rate of development of liver-related events. During the follow-up period, 13 patients developed liver-related events. Liver-related events included liver cancer, hepatic encephalopathy, esophago-gastric varices, ascites, and jaundice

In the present study, the cumulative incidence rates of liver cancer were 1.6% and 2.3% at the end of 5 and 10 years, respectively. The yearly incidence rate of liver-related events over the 10-year study period was 0.23%. Univariate analysis did not identify noninvasive parameters that correlated significantly with the incidence of liver cancer.

## Discussion

Previous reports have demonstrated that the stage of fibrosis in patients with NAFLD is one of the most important predictors for the incidence of the three complications described in this study (CVDs, malignancies [except for liver cancer], and liver-related events [10]. Our results confirmed the above results in which the multivariate analysis identified a FIB-4 index ≥ 2.67 as an advanced stage of fibrosis in addition to a significant and independent predictor of three complications. However, the present results concerning CVDs and malignancies (except for liver cancer) should be carefully interpreted due to the small number of events during the study period. FIB-4 index could be identified as the most powerful predictor of the three factors associated with fibrosis stage (platelet count), inflammation (AST, ALT), and age. The previous study was based on NAFLD patients who were diagnosed by US at a heath checkup in a tertiary hospital in Korea and demonstrated that a high FIB-4 index showed a strong association with the development of malignancies (except for liver cancer) [17]. However, a recent report based on adults in Sweden with NAFLD confirmed by biopsy indicated that a significant excess mortality risk was found across all stages of fibrosis associated with NAFLD, and this increased risk was primarily due to deaths from malignancies (except for liver cancer) [18]. Hence, the usefulness of the FIB-4 index for the prediction of the incidence of CVDs and malignancies (except for liver cancer) should be also evaluated in terms of the impact of not only the stage of fibrosis but also with respect to the age of the patient [19].

It is still unclear whether the impact of genetic factors, including the *PNPLA3* genotype, might affect CVDs in patients with NAFLD. The influence of the *PNPLA3* genotype on retinol metabolism might occur at the level of hepatic stellate cells, or hepatocytes lipid droplets could potentially play a role in NAFLD progression. The remodeling of specific lipids and retinol plays a pivotal role in NAFLD development and contributes to fat accumulation, inflammation, and fibrogenesis [20]. Previous Japanese report showed that it has been reported that levels of fasting plasma glucose and triglyceride were higher in patients with *PNPLA3* CC type [21]. Interestingly, in the present cohort, patients with CC type significantly indicated the higher rates of type 2 diabetes mellitus and hyperuricemia than those with non-CC type, respectively (type 2 diabetes mellitus; CC 47.4% versus non-CC 33.1%, *P* = 0.048; Chi-squared test) (hyperuricemia; CC 24.6% versus non-CC 11.0%, *P* = 0.010; Chi-squared test). The present findings support that there might be the tendency for patients with CC type to have more complications related to CVDs risk. However, mendelian randomization analysis recently indicated that the *PNPLA3* genetic variant might not be causally associated with the risk of CVDs. Among other genetic variants related to NAFLD, *TM6SF2* appears to be protective, whereas *MBOAT7* could favor venous thromboembolism [22, 23]. Furthermore, the previous Japanese report indicated that the *PNPLA3* GG type might affect development of liver-related events, including liver cancer [24]. The present study showed that the *PNPLA3* CC type might be one risk factor for CVD in patients with NAFLD. The differences in these results might be due to racial differences, study design, small number of events, collider bias, and differences in the impact of *PNPLA3* genotype on two complications (phenotype of CVDs or liver-related events). Further studies that matched the patients’ backgrounds, including racial background or ethnicity, should be performed to investigate the impact of genetic factors on the incidence of complications in patients with NAFLD.

Our results highlight the importance of the combination of the three risk factors (*PNPLA3* genotype, CKD, and FIB-4 index) for early prediction of CVDs in patients with NAFLD. Recent reports show that a high frequency of NAFLD could be observed among the patients with CKD and a possible association of NAFLD with the increase in CVDs among those patients [25]. The present study identified CKD as one risk factor for the incidence of CVDs in patients with NAFLD and agreed to this recent report [25]. The mechanism of the increase in the CVDs is still unknown in NAFLD patients with CKD. Further studies based on the large number of patients should be performed to investigate the impact of CKD on CVDs in patients with NAFLD.

Differences in malignancies (except for liver cancer) according to gender should be investigated. A previous report from Korea showed that male patients with NAFLD had a higher association of developing colon cancer and female patients developed breast cancer as malignancies other than liver cancer [17]. Overall, the present study identified the increase in colon cancer and particularly showed higher rates of lung cancer in males and colon cancer in females. The present study presents certain limitations. This discrepancy between the previous report and the present study could be due to differences in racial factors and the small number of events during the study period. Further studies with a larger number of NAFLD patients and longer follow-up periods should be performed to investigate malignancies (except for liver cancer) according to gender.

In conclusion, the present results highlight the importance of genetic factors and FIB-4 index on the incidence of complication in Japanese patients with histo-pathologically confirmed NAFLD, especially with respect to the incidence of CVDs. Early diagnosis based on the presence of one or more risk factor in addition to early treatment might improve the prognosis in patients with NAFLD. Further prospective and multi-center study based on the large number of patients should be performed to investigate the reliability of the present findings.

## Conclusions

Our results highlight the importance of the *PNPLA3* genotype and FIB-4 index ≥ 2.67 on the incidence of complications in Japanese patients with NAFLD, especially the incidence of CVDs.

## Data Availability

The datasets generated and/or analyzed in the present study are available from the corresponding author on reasonable request.

## Abbreviations

AST: Aspartate aminotransferase
ALT: Alanine aminotransferase
AUROC: Area under the receiver-operating characteristic curve
CI: Confidence interval
CKD: Chronic kidney disease
CVDs: Cardiovascular diseases
FIB-4 index: Fibrosis-4 index
HR: Hazard ratio
NAFLD: Non-alcoholic fatty liver disease
NAS: NAFLD activity score
NASH: Non-alcoholic steatohepatitis
PPV: Positive predictive value
NPV: Negative predictive value

## Declarations

### Disclaimers

This paper has not been published or presented elsewhere in part or in entirety, and is not under consideration by another journal.
